# RNA sequencing uncovers key players of cartilage calcification: potential implications for osteoarthritis pathogenesis

**DOI:** 10.1093/rheumatology/keae587

**Published:** 2024-10-21

**Authors:** Ilaria Bernabei, Elodie Faure, Julien Wegrzyn, Nicolas Bertheaume, Guillaume Falgayrac, Thomas Hugle, Sonia Nasi, Nathalie Busso

**Affiliations:** Service of Rheumatology, Department of Musculoskeletal Medicine, Lausanne University Hospital and University of Lausanne, Lausanne, Switzerland; Service of Rheumatology, Department of Musculoskeletal Medicine, Lausanne University Hospital and University of Lausanne, Lausanne, Switzerland; Service of Orthopedics, Department of Musculoskeletal Medicine, Lausanne University Hospital and University of Lausanne, Lausanne, Switzerland; University of Lille, CHU Lille, Univ. Littoral Côte d’Opale, ULR 4490 - MABLab- Adiposité Médullaire et Os, Lille, France; University of Lille, CHU Lille, Univ. Littoral Côte d’Opale, ULR 4490 - MABLab- Adiposité Médullaire et Os, Lille, France; Service of Rheumatology, Department of Musculoskeletal Medicine, Lausanne University Hospital and University of Lausanne, Lausanne, Switzerland; Service of Rheumatology, Department of Musculoskeletal Medicine, Lausanne University Hospital and University of Lausanne, Lausanne, Switzerland; Service of Rheumatology, Department of Musculoskeletal Medicine, Lausanne University Hospital and University of Lausanne, Lausanne, Switzerland

**Keywords:** OA, cartilage calcification genes, calciprotein particles, RNA sequencing

## Abstract

**Objective:**

OA is a joint disease linked with pathologic cartilage calcification, caused by the deposition of calcium-containing crystals by chondrocytes. Despite its clinical significance, the precise mechanisms driving calcification remain elusive. This study aimed to identify crucial players in cartilage calcification, offering insights for future targeted interventions against OA.

**Methods:**

Primary murine chondrocytes were stimulated with secondary calciprotein particles (CPP2) or left untreated (NT) for 6 h. Calcification was assessed by alizarin red staining. RNA was analysed by Bulk RNA sequencing. Differentially expressed (DE) genes were identified [cutoff: abs(LogFC)>1 and adjusted *P*-value < 0.05], and top 50 DE genes were cross-referenced with human OA datasets from previous studies (i.e. healthy vs. OA cartilage, or undamaged vs. damaged cartilage). RNA from NT and CPP2-stimulated primary human OA chondrocytes were used to validate genes by qPCR.

**Results:**

CPP2 induced crystal formation by chondrocytes and significantly modulated 1466 genes. Out of the top 50 DE genes in CPP2, 27 were confirmed in published OA cartilage datasets. Of those genes, some are described in calcification and/or OA (*Errfi1*, *Ngf*, *Inhba*, *Col9a1*). Two additional ones (*Rcan1*, *Tnfrsf12a*) appear novel and interesting in the context of calcification and OA. We validated modulation of these six genes in calcifying human chondrocytes from five patients. Ultimately, we unveiled two distinct gene families modulated by CPP2: the first comprised cytoskeletal genes (*Actb*, *Tpm1*, *Cfl1*, *Tagln2*, *Lmna*), while the second encompassed extracellular matrix genes (*Fmod*, *Sparc*, *Col9a1*, *Cnmd*).

**Conclusion:**

CPP2 modulates genes in chondrocytes that could represent new targets for therapeutic interventions in OA.

Rheumatology key messages

- Secondary calciprotein particles (CPP2) trigger calcification and OA-related genes in murine and human chondrocytes.
- Ongoing calcification (CPP2) and established calcification (hydroxyapatite crystals) modulate common genes.
- Calcification modulates cytoskeleton and extracellular matrix-related genes.

## Introduction 

OA is the most prevalent degenerative joint disease characterized, at late stage, by articular cartilage calcification (also called mineralization), cartilage damage, synovitis and subchondral bone remodelling [1]. Risk factors include age, obesity, joint trauma and heredity. Current therapies focus on symptom relief, such as anti-inflammatory drugs, or, as final resolution, joint replacement surgery, both often associated with complications [2]. The absence of a definitive solution, coupled with a progressively ageing population, contributes to the increasing social and economic burden of this disease. Therefore, understanding the mechanisms driving OA is crucial for advancing effective and targeted interventions.

Pathologic calcification, a hallmark of OA, correlates with histological cartilage damage and radiographic score for disease severity [3–6]. The production of the two main crystal families, basic calcium phosphate (BCP) and calcium pyrophosphate (CPP), is regulated by a complex machinery composed of enzymes and transporters that modulate the levels of pyrophosphate (PP_i_), phosphate (P_i_) and calcium ions (Ca^2+^) [7]. Various types of BCP crystals have been identified in cartilage, notably carbonated hydroxyapatite (CA), hydroxyapatite (HA) and octacalcium phosphate [8, 9]. Pathologic calcification is typically accompanied by reactive oxygen species (ROS) production [10] and chondrocyte apoptosis [11]. Next, once formed, crystals can amplify these effects [7], ultimately leading to cartilage damage, via release of matrix-degrading enzymes (MMPs).

In recent years, new players in serum calcium homeostasis were discovered, called calciprotein particles [12]. They are colloidal nanoparticles formed by the binding of circulating P_i_, Ca^2+^ and fetuin-A. These further aggregate in small spherical primary calciprotein particles (CPP1). Over time, CPP1 spontaneously convert to secondary calciprotein particles (CPP2), bigger and needle-shaped, known to be responsible for the progression of pathologic calcification in tissues. The transition between CPP1 and CPP2 can be used as a marker for the propensity to calcify of a patient [13]. CPP2 can also be synthetically formed *in vitro* [14], and their incubation with cells has been shown to induce calcium crystal deposition by vascular smooth muscle cells and chondrocytes [14, 15]. However, the effects of CPP2 on chondrocytes have not been described.

In the present study, we aimed to explore the transcription profile of murine chondrocytes stimulated with CPP2. We discovered new modulated genes by CPP2 and potential networks involved in pathologic calcification, providing possible targets for future therapies.

## Methods

### Murine chondrocytes isolation

Knees of 4- to 7-day newborn mice were dissected and chondrocytes were isolated after treatment with Liberase™ (Roche) overnight, cell number was expanded in DMEM high glucose + 10% FBS (fetal bovine serum) and used at passage 2 [15].

### Human chondrocytes isolation

Human chondrocytes were isolated from the cartilage of five OA patients undergoing knee replacement surgery (two females, age 70 ± 2 years, three males, age 74 ± 4.2 years). Undamaged cartilage was selected based on macroscopically intact areas. After digestion overnight with Liberase™ (Roche) as previously described [15], cells were amplified in DMEM high glucose + 10% FBS and used at passage 2. Samples were obtained upon patient informed consent and with the approval of the Hospital Ethical Committee (n°2024–00117).

### Chondrocytes stimulations

Triplicates of chondrocyte monolayers (1.2 × 10^5^/cm^2^) were cultured in non-treated medium (NT: DMEM high glucose), in secondary calciprotein particles medium (CPP2: DMEM high glucose + 10% CPP2) or in the presence of HA crystals for 6 h. CPP2 were prepared as previously described [15]. Both HA and CPP2 were used at final concentration equivalent to 25 µg/ml calcium.

### Alizarin red staining

Crystal formation was analysed at the end of stimulation by 0.5% Alizarin red staining. Crystals were dissolved in 10% cetylpyridinium chloride and absorbance was measured by spectrophotometry (570–630 nm) [16].

### Raman analysis

Pellets obtained from chondrocyte monolayers were spread on quartz microscope slide. To capture the crystals’ Raman spectra, we used a Raman microspectrometer LabRAM HR800 (HORIBA JY, France), with a XYZ motorized stage and a 785 nm laser diode (power 10 mW at the sample). The acquisitions were performed using an Olympus × 100 objective (N.A. = 0.9). Acquisition time was 30 s averaged twice (60 s total acquisition time) and the spectral domain was 350–1700 cm^−1^. The spectral resolution was 4 cm^−1^. The acquired spectra underwent processing using a Savitzky−Golay smoothing filter (width: 3; polynomial order: 2) with a polynomial baseline correction (degree 4).

### RNA extraction and cDNA synthesis and qRT-PCR analysis

RNA was isolated using the RNA Clean and Concentrator kit from Zymoresearch, followed by reverse transcription with Superscript II, Invitrogen. Real-time PCR with gene-specific primers was conducted on the LightCycler480^®^ system from Roche Applied Science (see Supplementary Table S1, available at *Rheumatology* online for the full list of primers, available at *Rheumatology* online). Data normalization was performed against Gapdh reference gene, and the fold change of gene expression was calculated relative to the specified control.

### Bulk RNA barcoding and sequencing (BRB-seq)

Stimulated murine primary chondrocytes were lysed following MERCURIUS™ Extraction-free DRUG-seq kit, provided by Alithea Genomics SA (Lausanne, Switzerland). Lysates were frozen at −80°C and submitted to Alithea for library preparation [17]. The generation of highly multiplexed 3′-end BRB-seq libraries was performed using the MERCURIUS™ DRUG-seq library preparation kit for Illumina and following the manufacturer’s manual (Alithea Genomics, #10841). All libraries were sequenced on an Illumina Novaseq 6000.

### Alignment, quantification and differential expression analysis

In this study, STARsolo v2.7.9a was used to align and quantify the raw barcodes RNA-seq reads against the GRCm38.102 (mouse) genome. The parameters ‘--soloUMIdedup NoDedup 1MM_Directional’ and ‘--quantMode GeneCounts’ [18] were used to generate raw and UMI-deduplicated count matrices, opting for non-deduplicated counts for subsequent analyses. For revealing modulated genes, we employed the DESeq2 tool from the Bioconductor R package [19], implementing criteria of abs(LogFC)>1 and FDR (false discovery rate) < 0.05 for statistically significant gene selection. Results can be found in Supplementary Table S2, available at *Rheumatology* online. Furthermore, we analysed protein–protein interactions using the online tool STRING (v12.0) and selected *Homo sapiens* as the organism of interest [20]. Genes with ≥5 interactions with other genes were identified as hubs. Finally, differentially expressed genes were compared between datasets in RStudio (RStudio Team (2021). RStudio: Integrated Development Environment for R. RStudio V.1.4, PBC, Boston, MA).

### Meta-analysis

We performed a meta-analysis across eight human OA datasets available in the literature, where FC or log2(FC) and *P*-value were given (details on the chosen datasets in Supplementary Table S3, available at *Rheumatology* online). Four datasets contained a total of 86 patients’ samples of damaged cartilage compared with undamaged, while four datasets contained a total of 43 OA patients’ cartilage samples compared with 40 normal or healthy cartilage samples. Analysis was performed in RStudio. All values can be found in Supplementary Table S4, available at *Rheumatology* online.

### Statistical analysis

Values are expressed as mean ± SD. Variation between paired data sets was evaluated using parametric (paired *t*-test) or non-parametric (Wilcoxon) Student’s *t*-test. For unpaired data sets, we used the Mann–Whitney *U* test. Differences were considered statistically significant at ∗*P* < 0.05, ∗∗*P* < 0.01, ∗∗∗*P* < 0.001, ∗∗∗∗*P* < 0.0001. Graphs were made with GraphPad Prism software Version 10 (GraphPad).

### Ethical approval

The studies involving human participants were reviewed and approved by the Centre Hospitalier Universitaire Vaudois, Lausanne, Switzerland. The patients provided their written informed consent to participate in this study. Murine cells collection was approved by the ‘Service de la Consommation et des Affaires Vétérinaires du canton de Vaud’ Switzerland, animal authorization n° 3737b.

## Results

### CPP2 trigger calcification and OA-related genes in murine chondrocytes

**Figure 1.**
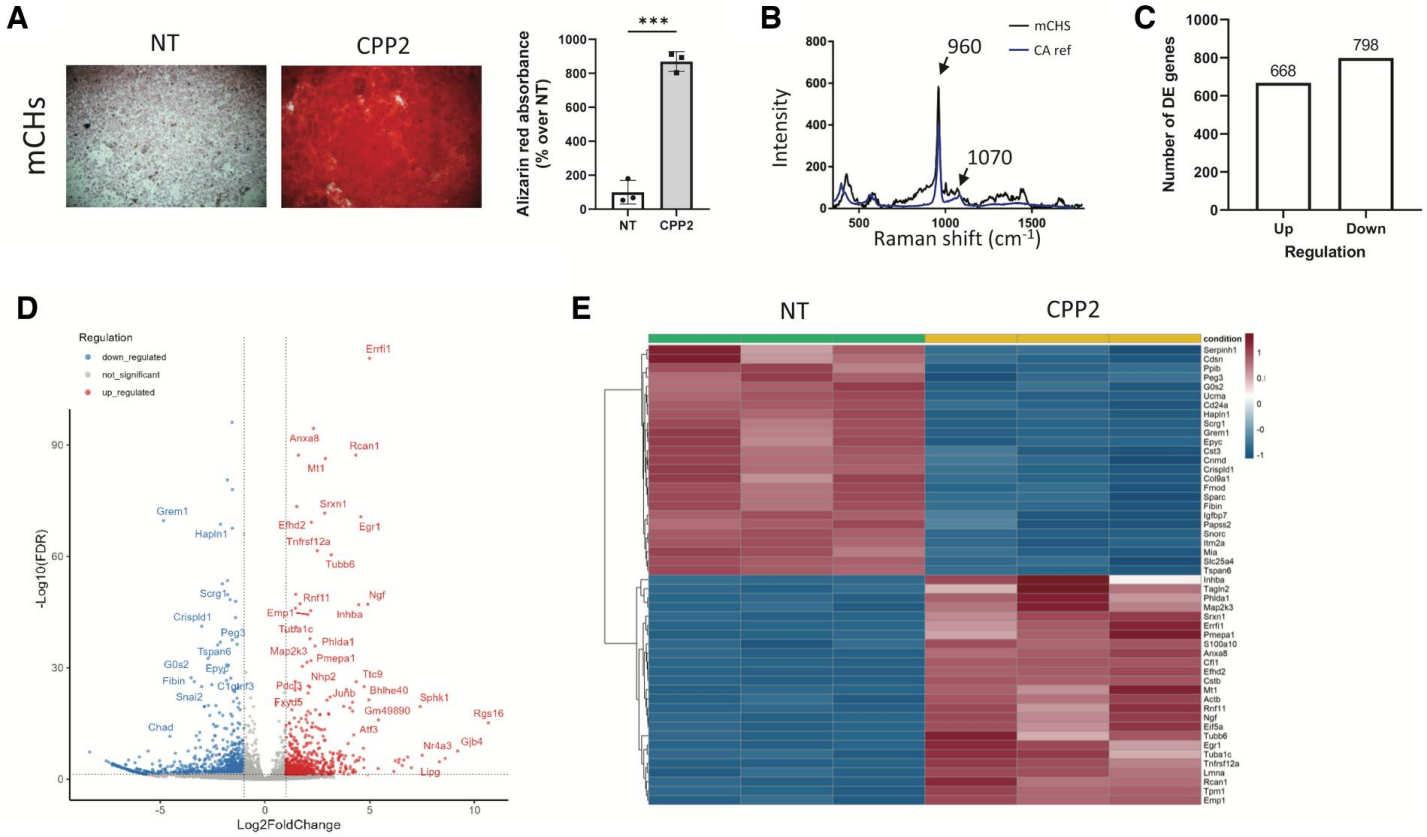
BRB-seq analysis of calcifying primary murine chondrocytes. (**A**) Alizarin red staining of primary mCHs stimulated with CPP2 for 6 h (left), and its quantification (results expressed in % over NT, right). Unpaired *t*-test, *n* = 3. (**B**) Raman spectrum of crystals produced after 6 h chondrocytes stimulation with CPP2. Peaks at 960 and 1070 cm^−1^ (black line) correspond to carbonated hydroxyapatite reference (CA, blue line). (**C**) Up- and downregulated genes from DE analysis from cultures performed in triplicates with CPP2 and NT mCHs (abs(logFC)>1 and adjusted *P*-value < 0.05). (**D**) Volcano plot of DE genes from (C). (**E**) Heatmap of the 50 most DE genes from (C). BRB: Bulk RNA barcoding and sequencing; mCHs: murine chondrocytes; NT: non-treated medium

CPP2 stimulation of mouse primary chondrocytes (mCHs) for 6 h promoted eight times more crystal formation by chondrocytes compared with the NT condition (Fig. 1A). We further characterized the calcium-containing crystals by Raman microspectroscopy which revealed that those produced upon CPP2 stimulation were exclusively CA crystals, as indicated by peaks at 960 and 1070 cm^−1^ [8] (Fig. 1B).

By BRB-seq, we first determined that CPP2-stimulated chondrocytes and NT chondrocytes showed a high degree of expression correlation (Supplementary Fig. S1A, available at *Rheumatology* online). Second, we identified by differential expression (DE) analysis 668 significantly up- and 798 downregulated genes in mCHs (Fig. 1C and D and Supplementary Table S2, available at *Rheumatology* online). First, we determined the top 50 DE genes (25 up- and 25 downregulated; Fig. 1E and Tables 1 and 2). Next, we compared the expression of the top 50 genes with RNA datasets available from previous publications (Supplementary Table S3, available at *Rheumatology* online). These studies compared the gene expression between OA cartilage and healthy cartilage, or between damaged and undamaged OA cartilage, where Log(FC) and *P*-value were provided. We confirmed that 27 (20 up- and 7 downregulated) of our CPP2-modulated genes were similarly regulated in published human OA datasets (Table 1 and Supplementary Table S4, available at *Rheumatology* online), while the remaining 23 genes were not confirmed (Table 2 and Supplementary Table S4, available at *Rheumatology* online).

**Table 1.** 27 genes from the 50 most DE genes in mCHs CPP2 vs. NT, confirmed by externally available datasets of OA cartilage

Gene name	Log2FC	Adjusted *P*-value	Confirmed dataset n°	Experimental evidence in OA	Experimental evidence in calcification
*Errfi1*	4.99	4.75E-114	8	Yes	No
*Ngf*	4.91	8.04E-48	1, 2, 4, 5, 8	Yes	Yes
*Inhba*	4.47	1.07E-47	1, 3, 7, 8	Yes	No
*Rcan1*	4.33	5.57E-88	4, 5, 7, 8	No	No
*Tubb6*	3.17	3.72E-61	7	No	No
*Tnfrsf12a*	2.50	3.23E-62	1, 3, 4, 5, 6, 7	No	No
*Phlda1*	2.40	1.40E-36	6, 7	No	Yes
*Anxa8*	2.32	3.54E-95	1, 3, 4	Yes	No
*Efhd2*	2.21	6.76E-70	6, 7	No	No
*Pmepa1*	2.19	1.19E-32	8	No	No
*Tuba1c*	2.15	1.46E-38	6, 7	Yes	No
*Emp1*	2.04	4.57E-45	6, 7	No	No
*Map2K3*	2.00	3.32E-32	7, 9	No	No
*Tagln2*	1.78	4.04E-31	5, 6	No	No
*Lmna*	1.67	5.90E-48	6, 7	No	No
*Cfl1*	1.52	4.07E-74	6, 7	No	No
*S100a10*	1.48	9.97E-42	5, 6	No	Yes
*Tpm1*	1.47	1.68E-50	6, 7	No	No
*Eif5a*	1.45	9.31E-47	7	No	No
*Cstb*	1.44	4.81E-27	6, 7	No	No
*Grem1*	−4.84	2.46E-70	1, 8	Yes	Yes
*G0s2*	−3.53	4.97E-28	5, 8	Yes	No
*Crispld1*	−3.02	6.94E-42	1, 2, 3, 8	No	No
*Col9a1*	−1.56	3.33E-38	1, 2, 4, 8	Yes	Yes
*Ucma*	−1.55	1.05E-78	1	Yes	Yes
*Serpinh1*	−1.41	3.16E-44	8	Yes	Yes
*Cst3*	−1.33	4.73E-37	8	No	Yes

Comparison between OA cartilage vs. healthy or between OA damaged vs. undamaged cartilage.

mCHs: murine chondrocytes; CPP: calciprotein particles; NT: non-treated medium.

**Table 2.** Twenty-three genes from the most 50 DE genes in mCHs CPP2 vs. NT, not confirmed in externally available datasets

Gene name	Log2FC	Adjusted *P*-value	Experimental evidence in OA	Experimental evidence in calcification
*Egr1*	4.58	2.26418E-71	Yes	Yes
*Mt1*	2.88	4.72913E-87	Yes	No
*Srxn1*	2.85	2.29612E-72	No	No
*Rnf11*	2.18	4.11714E-46	No	Yes
*Actb*	1.60	5.57442E-88	No	No
*Fibin*	−3.37	5.79748E-27	No	No
*Epyc*	−2.72	3.201E-33	Yes	No
*Tspan6*	−2.26	7.63576E-37	No	No
*Peg3*	−2.13	1.27044E-37	No	No
*Hapln1*	−2.12	2.25744E-69	Yes	No
*Scrg1*	−2.04	2.65055E-53	No	No
*Igfbp7*	−1.96	1.99203E-29	Yes	Yes
*Mia*	−1.84	2.70358E-27	Yes	Yes
*Sparc*	−1.83	1.61484E-31	No	Yes
*Cd24a*	−1.79	2.70932E-81	No	No
*Cnmd*	−1.79	3.31507E-54	Yes	No
*Fmod*	−1.79	2.12874E-50	Yes	Yes
*Cdsn*	−1.74	2.23488E-31	No	No
*Slc25a4*	−1.67	4.46506E-49	No	No
*Papss2*	−1.63	6.82114E-28	Yes	No
*Snorc*	−1.57	7.92424E-97	No	No
*Itm2a*	−1.56	2.64509E-68	No	No
*Ppib*	−1.40	1.32809E-48	No	No

mCHs: murine chondrocytes; CPP: calciprotein particles; NT: non-treated medium.

Moreover, we identified by manual search of the literature that 24 out of the top 50 DE genes were already described in calcification and/or OA (Tables 1 and 2): 5 genes in calcification only (*Phlda1*, *S100a10*, *Cst3*, *Rnf11*, *Sparc*); 10 genes in OA only (*Errfi1*, *Inhba*, *Ana8*, *Tuba1c*, *G0s2*, *Mt1*, *Epyc*, *Hapln1*, *Cnmd*, *Papss2*); 9 genes in both calcification and OA (*Ngf*, *Grem1*, *Col9a1*, *Ucma*, *Serpinh1*, *Egr1*, *Igfbp7*, *Mia*, *Fmod*). For the highest upregulated genes, the range of modulation spanned from 20- to 32-fold, while for the most downregulated genes, the range was 3- to 29-fold.

**Figure 2.**
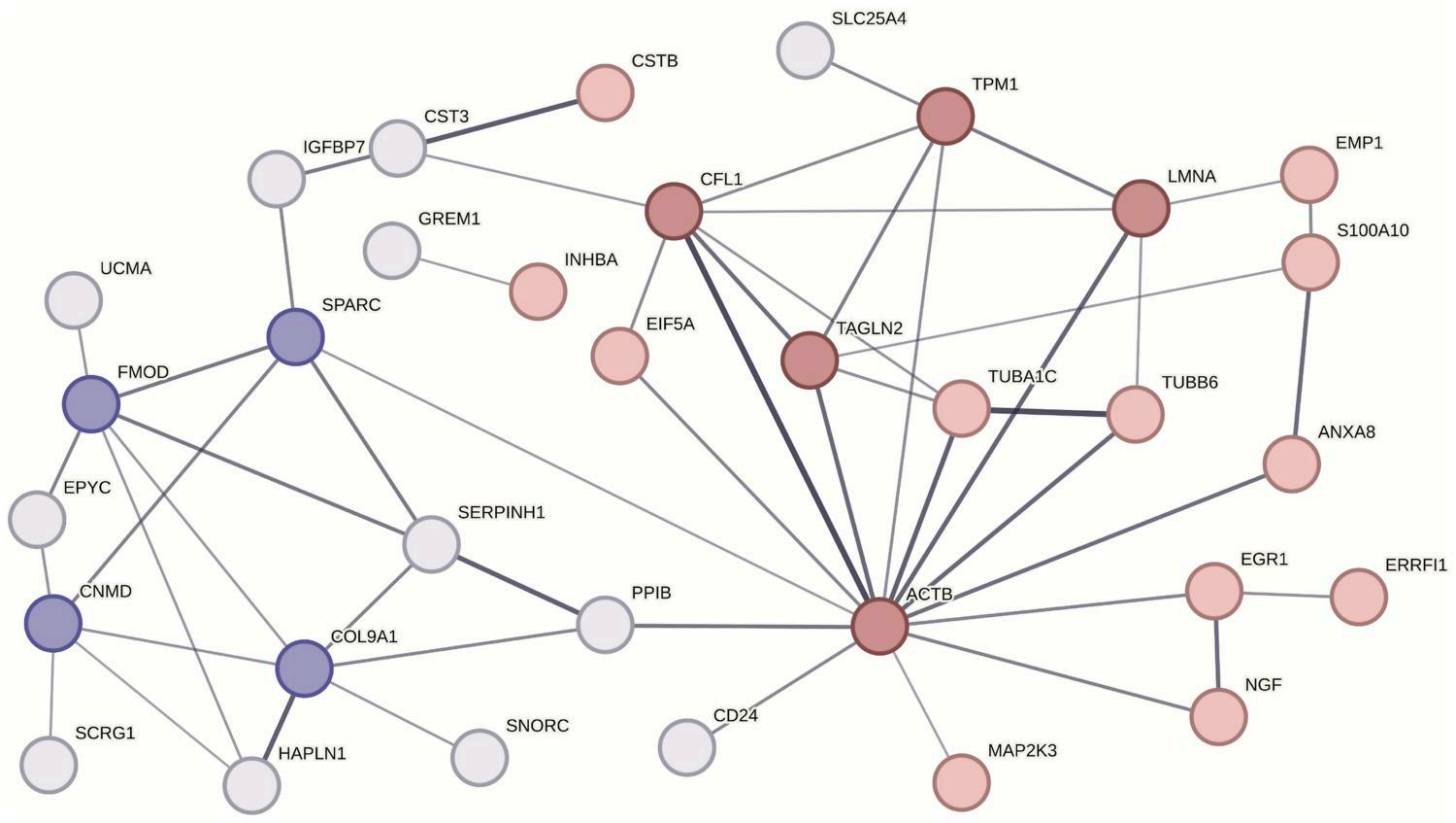
Graphical representation of protein-to-protein interactions of 33 of the top 50 DE genes in mCHs CPP2 vs. NT, using STRING database (v.12.0). Nodes represent proteins, edges represent protein–protein associations. The thickness of the edges indicates the strength of data support (Confidence: low 0.15, medium 0.4, high 0.7, highest 0.9). Red = upregulated genes; blue = downregulated genes. Hub genes, identified as genes with interaction with at least 5 other genes, are marked in dark red for upmodulated genes and dark blue for downmodulated ones. mCHs: murine chondrocytes; NT: non-treated medium

Finally, to explore interactions between proteins encoded by the top 50 DE genes, we used the online tool STRING (v.12.0). We identified 33 genes that have a significant degree of connection (*P*-value = 1.67 × e^−15^). Most interestingly, these genes segregated into two groups: upregulated genes (Fig. 2 in red) interacting mostly with other upregulated genes, and downregulated genes (Fig. 2 in blue) interacting mostly with other downregulated genes. In particular, we identified five hubs amongst the upmodulated genes (*Actb*, *Tpm1*, *Cfl1*, *Tagln2*, *Lmna*, Fig. 2 in dark red) and four hubs amongst the downmodulated ones (*Fmod*, *Sparc*, *Col9a1*, *Cnmd*, Fig. 2 in dark blue).

### Ongoing calcification (CPP2 stimulation), and established calcification (HA crystals), modulate common genes

We showed that CPP2 stimulation led to CA production by chondrocytes (Fig. 1A and B). We wished to investigate if CPP2 is the direct trigger of gene modulation, or if the modulation occurs via CPP2-induced HA. To do this, we performed RNA sequencing on primary murine chondrocytes stimulated with HA crystals.

**Figure 3.**
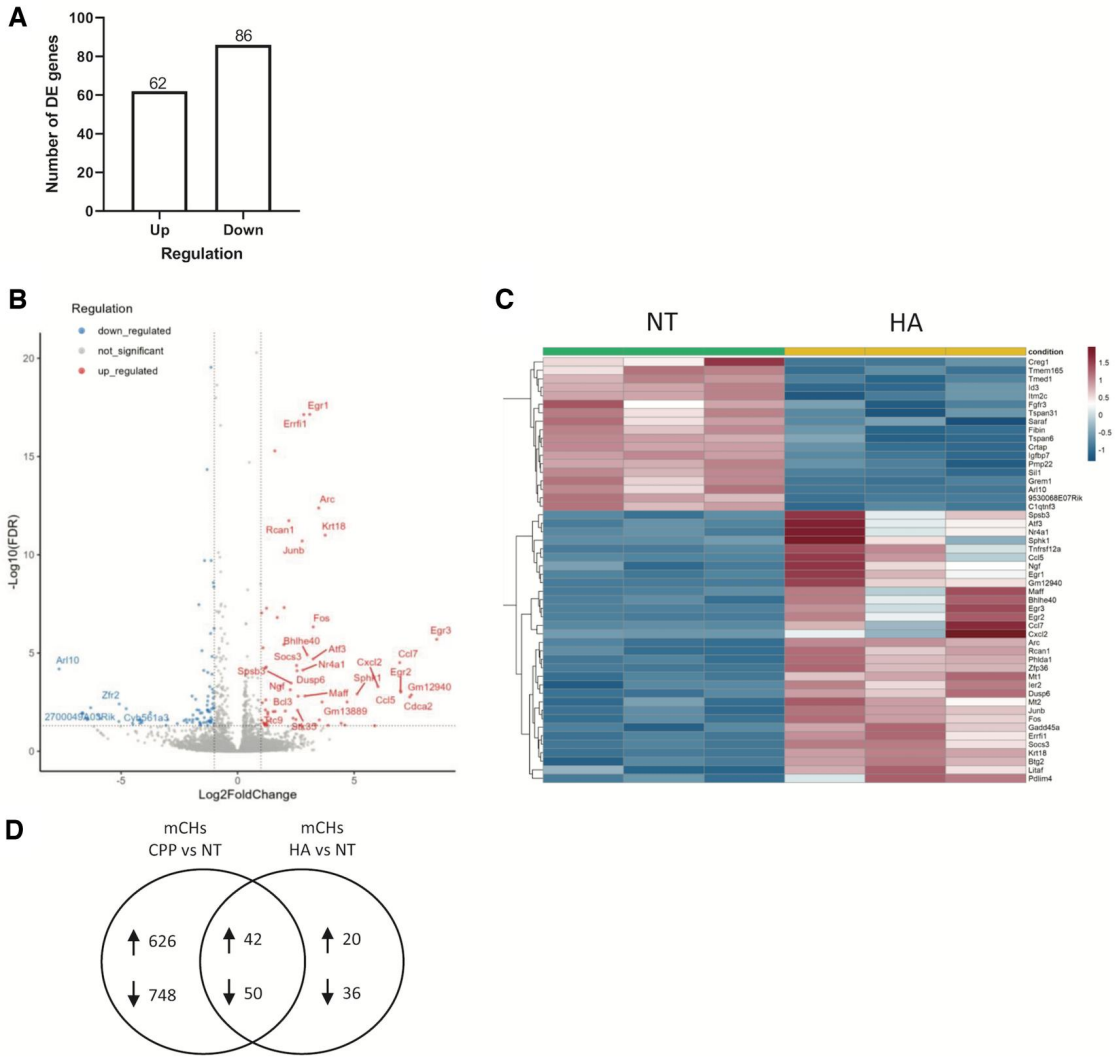
BRB-seq analysis of HA crystal-stimulated primary murine chondrocytes. (**A**) Up- and downregulated genes from DE analysis of HA vs. NT mCHs (abs(logFC)>1 and adjusted *P*-value < 0.05). (**B**) Volcano plot of DE genes from (A). (**C**) Heatmap of the top 50 DE genes from (A). (**D**) Venn diagram representing the number of common up- and downregulated genes between mCHs CPP vs. NT and HA vs. NT. BRB: Bulk RNA barcoding and sequencing; HA: hydroxyapatite; NT: non-treated medium; mCHs: murine chondrocytes; CPP: calciprotein particles

We first confirmed the degree of expression correlation between HA and NT samples and their significant differential expression (Supplementary Fig. S1B, available at *Rheumatology* online). Next, DE analysis revealed 62 significantly upregulated and 86 downregulated genes between HA and NT cells (Fig. 3A and B and Supplementary Table S2, available at *Rheumatology* online). Of those, the top 50 DE genes were determined (32 up- and 18 downregulated; Fig. 3C). We next searched if there were genes modulated by both CPP2 and HA (Fig. 3D, Supplementary Table S5, available at *Rheumatology* online). We found 42 commonly upregulated genes and 50 commonly downregulated genes. Amongst these 92 genes, we confirmed that 30 up- and 12 downmodulated genes were similarly regulated in previously published human OA datasets (Supplementary Table S4, available at *Rheumatology* online). Taken together, these results highlight that CPP2 modulate genes via both HA-dependent and independent ways.

### Validation of CPP2-modulated genes in human OA chondrocytes

**Figure 4.**
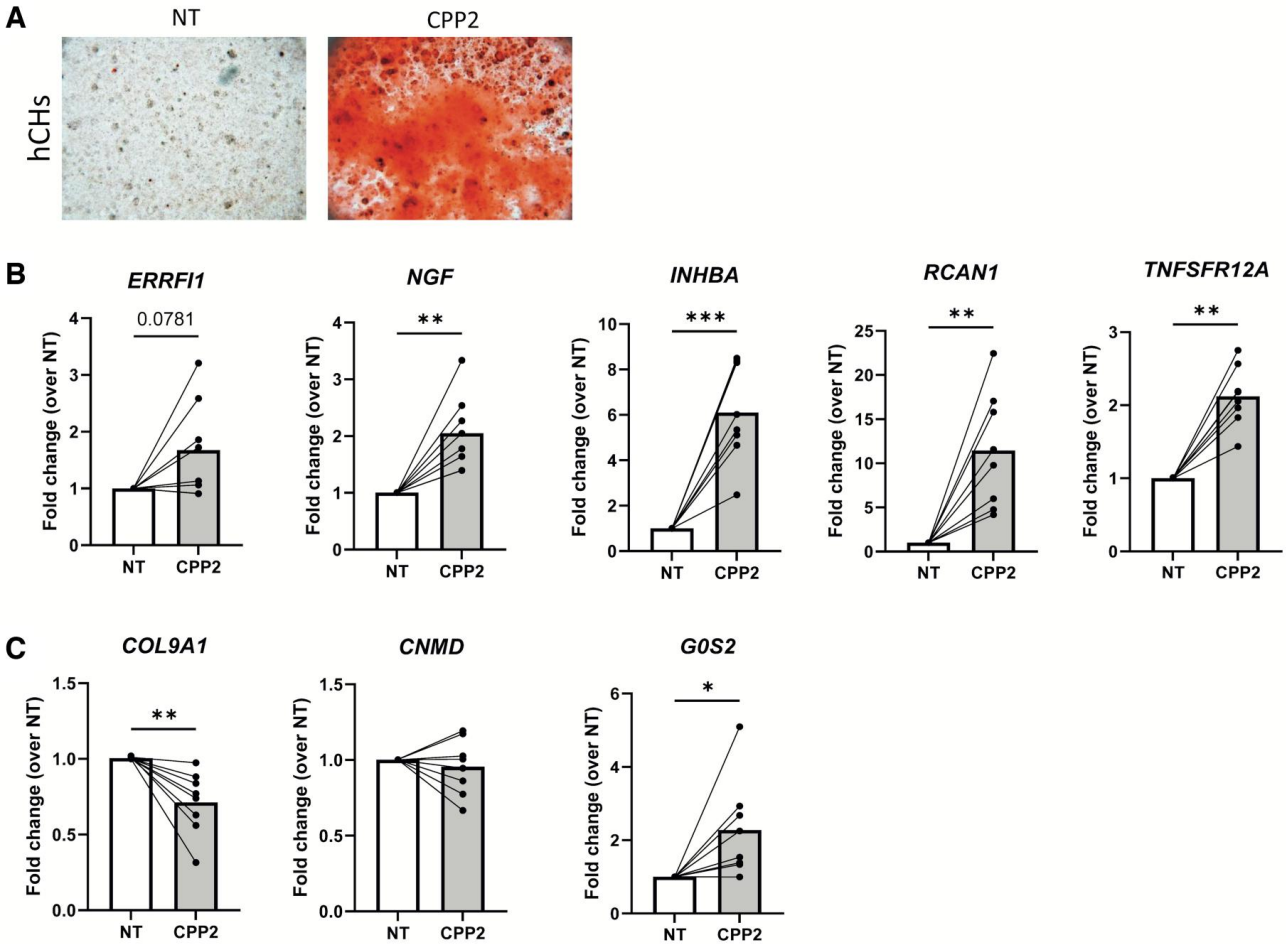
Confirmatory results in primary human chondrocytes. (**A**) Alizarin red staining of primary hCHs stimulated with CPP2 or NT for 6 h. Representative image of one out of 5 patients. (**B**) qRT-PCR analysis was carried out to assess expression of upmodulated genes. (**C**) qRT-PCR analysis of downregulated genes. *n* = 8 human patients’ chondrocytes. *t*-Test paired for all genes. hCHs: human chondrocytes; NT: non-treated medium

We next wanted to validate CPP2-gene modulation in primary human chondrocytes from undamaged OA cartilage of five patients. As observed in murine chondrocytes, CPP2 at 6 h induced massive calcification (Fig. 4A). We next selected eight genes (five up and three down) from the top differentially expressed genes in Table 1. We confirmed that *ERRFI1*, *NGF*, *INHBA*, *RCAN1* and *TNFSFR12A* were also upmodulated in calcifying human chondrocytes, although *ERRFI1* did not reach significance (Fig. 4B). We also corroborated downmodulation of *COL9A1*, while we could not reproduce *CNMD* and *G0S2* modulation (Fig. 4C).

## Discussion

This study is the first comprehensive analysis of gene expression changes occurring during crystal formation by chondrocytes. Amongst the top 50 DE genes modulated by CPP2 we identified not only genes already known in calcification and/or OA, but also novel genes and gene networks that could open new investigative avenues in OA pathogenesis.

Of the genes already known in calcification, CPP2 induced upregulation of *Phlda1* and *S100a10*, and downregulation of *Cst3*. *PHLDA1* knockdown in vascular smooth muscle cells and in vessels reduced HA deposition, via inhibited uptake of P_i_ and RUNX2 activity [21]. S100A10, a calcium-binding protein, translocated on ALP-rich matrix vesicles during mineralization [22], and its incubation with bone marrow-derived stem cells significantly increased mineralization [23]. CST3 expression increased in aged mice in and around chondrocytes located in calcified cartilage [24].

Amongst the genes with experimental evidence in OA, we found upregulated *Errfi1* and *Inhba* and downregulated *G0s2*. Cartilage-specific overexpression of *Errfi1* in mice led to accelerated OA [25]. INHBA was found to be more secreted by human OA cartilage explants compared with control samples [26], and its gene was highly expressed in articular cartilage of OA mice [27]. *G0s2* was significantly downregulated in human [28] and murine [29] OA cartilage, with respect to healthy cartilage control.

As mentioned above, we also found nine genes already described in both calcification and OA. We found induced *Ngf* by CPP2, previously reported increased in human OA cartilage [30], involved in glycosaminoglycan loss [31] and in crystal formation by chondrocytes [32]. CPP2 negatively regulated *Grem1* and *Col9a1*. Expression of *GREM1* inversely correlated with OA severity in human cartilage [33]. *Col9a1* knock-out in mice caused spontaneous OA [34] and led to accelerated ossification of mice femoral heads [35], while its expression was reduced in human OA cartilage [36].

Of relevance, we also identified new genes whose role has yet to be verified in calcification or OA, namely *Rcan1* and *Tnfrsf12a*. *Rcan1* is a negative endogenous regulator of calcineurin 1, whose signalling regulates osteoblast differentiation [37] and was found upregulated in OA epigenomic and transcriptomic datasets [38]. *Tnfrsf12a* is a receptor for TNF-related inducer of apoptosis and is involved in age-associated pathological changes in skeletal muscle and other organs [39].

Most interestingly, from our BRB-seq on chondrocytes, we established that two main families of genes were modulated by CPP2: the first comprised cytoskeletal genes, while the second encompassed extracellular matrix genes. Regarding the cytoskeleton family, we found significant upregulation of genes related to microfilaments (actin), microtubules (tubulin) and intermediate filaments. CPP2 induced β-actin (*Actb*) and its regulators, including *Efhd2* (Ca^2+^ binding actin protein), *Cfl1* (actin-binding protein), *Tagln2* (actin cross-linker) and *Tpm1* (regulator of actin filaments) [40]. Moreover, we found upregulation of tubulin-β6 (*Tubb6*) and *-α1c* (*Tuba1c*) [40], as well as increased expression of *Lmna*, encoding for an intermediate filament of the nuclear lamina [40]. We were able to confirm the upregulation of all these genes in previously published human OA datasets, except for *Actb*. Previous OA studies showed that distribution and intensity patterns of cytoskeletal elements are altered by mechanic load or pro-inflammatory cytokines (TNF-α, -β1, and IL-1β) [41]. The microtubule assembly inhibitor colchicine reduced mineralization in bone-like cells [42], and blocked endocytosis of crystals (monosodium urate crystals and CPP crystals [43], but no evidence for BCP crystals so far). Another study suggested that upregulation of tubulin-β6 is required during differentiation of chondrocytes towards a hypertrophic calcifying phenotype [44]. Future experiments are required to investigate the relationship between cytoskeletal changes and the calcification process. Concerning the extracellular matrix family of genes impacted by CPP2 in chondrocytes, we revealed decreased expression of proteoglycans, *Fmod*, *Snorc* and *Epyc*, and proteoglycan-interacting proteins, *Hapln1* and *Papss2* [40]. Next, we found decreased collagen-related genes, namely *Col9a1*, *Serpinh1*, *Ppib*, *Ucma* and *Sparc* [40]. These findings are in line with overall diminished ECM (extracellular matrix) synthesis by chondrocytes during OA [45].

Finally, as we identified that CPP2 induced HA crystal production, we performed RNA sequencing of murine chondrocytes stimulated with HA. We found a smaller number of significant DE genes (62 up- and 86 downregulated) compared with CPP2-stimulated cells (668 up- and 798 downregulated). Interestingly, the majority of HA-modulated genes were in common with those modulated by CPP2 (42 out of 62 upregulated and 50 out of 86 downregulated). Amongst these common genes, we found several calcification- and/or OA-related genes, namely *Phlda1*, *Errfi1*, *Ngf* and *Grem1*, as well as the two novel genes, *Rcan1* and *Tnfsfr12a*. However, HA did not induce any cytoskeleton-related gene that we found increased by CPP2, nor diminished any of the genes encoding ECM proteins. These interesting findings may suggest that, at 6 h, chondrocytes in the process of active calcification undergo intracellular (cytoskeleton) and extracellular (ECM) rearrangement. By contrast, already formed crystals affect only intracellular pathways [7]. Although we found CA as the calcium-containing crystal produced by murine chondrocytes, several other BCP and CPP crystals have been described in OA cartilage [7]. Therefore, we plan future studies investigating the effect of different crystal types on chondrocytes.

The limitations of this study are: (i) all experiments were performed at 6 h of stimulation, which might conceal the modulation of other genes that happen at different time points. (ii) We focused our analysis on the top 50 DE genes, but we cannot exclude that other DE genes can play a role in calcification and/or OA. (iii) Our work is based on RNA transcription that does not always reflect protein expression and activity. For example, the cytoskeleton function depends mainly on the enzymatic activity on the filament elongation and termination [41], aspects that may remain undetected in this type of study. Nevertheless, we intend to validate our observations at the protein level through future investigations.

Overall, the transcriptomic profile of CPP2-stimulated chondrocytes revealed that active calcification modulates the expression of known genes in the context of calcification and/or OA (*Errfi1*, *Ngf*, *Inhba*, *Col9a1*), as well as unknown genes (*Rcan1*, *Tnfrsf12a*). Importantly, we validated the modulation of these genes in chondrocytes from five OA patients, thus strengthening their potential contribution to the pathogenesis of OA. Moreover, we have shown that chondrocytes producing crystals exhibited upregulation of cytoskeletal-related genes and downregulation of ECM-related genes. In conclusion, our study highlights the role of pathological calcification in OA cartilage and identifies potential key players in this process. Further mechanistic and detailed studies are needed to determine which of these targets could form the basis for new therapies and to explore potential strategies for their development.

## Supplementary Material

keae587_Supplementary_Data

## Data Availability

All data are displayed in the manuscript. Raw data are available upon request to the corresponding author.
