# Supplementary material for: RNA sequencing uncovers key players of cartilage calcification: potential implications for osteoarthritis pathogenesis
Source: Rheumatology (Oxford). 2024 Oct 21;64(5):3151–9. doi: 10.1093/rheumatology/keae587 (PMC12048056; doi:10.1093/rheumatology/keae587)
Supplement: keae587_Supplementary_Data [file keae587_supplementary_data.zip › keae587_Supplementary_Data/rhe-24-0644-File007.docx]

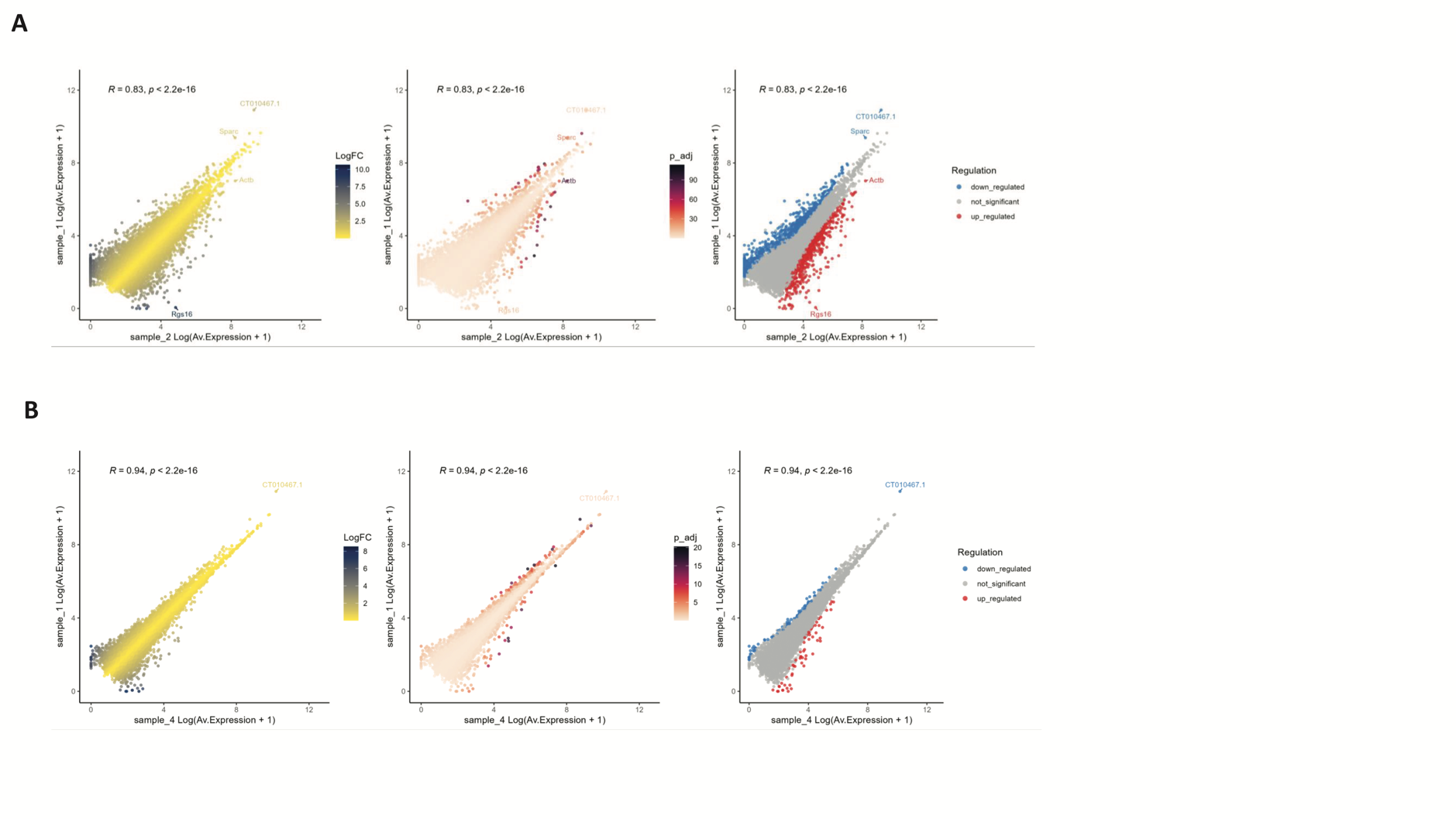


Supplementary Figure S1. Quality control of BRB-seq analysis of CPP2- and HA-stimulated primary murine chondrocytes
